# Comparison of serodiagnosis methods for community-acquired *Mycoplasma pneumoniae* respiratory tract infections in children

**DOI:** 10.1097/MD.0000000000034133

**Published:** 2023-07-21

**Authors:** Mengyang Liu, Ke Meng, Jun Jiang, Li Zhang, Shiying Sun

**Affiliations:** a Department of Clinical Laboratory, The Affiliated Hospital of Qingdao University, Qingdao, P.R. China; b Department of Physical Examination of Qilu Hospital (Qingdao), Shandong University, Qingdao, P.R. China; c Department of Immunology, School of Basic Medicine, Qingdao University, Qingdao, P.R. China.

**Keywords:** chemiluminesence immunoassay, indirect immunofluorescence assay, *Mycoplasma pneumoniae*, passive particle agglutination

## Abstract

This study aimed to evaluate the diagnostic value of chemiluminescence immunoassay (CLIA), passive particle agglutination (PPA), and indirect immunofluorescence assay (IFA) in detecting *Mycoplasma pneumoniae* infection in children. Serum samples from 165 children with acute community-acquired respiratory tract infections were examined using CLIA, PPA, and IFA, and consistency coefficient, specificity, and sensitivity were analyzed. Compared with the PPA (titer ≥ 1:160), the consistency coefficients of the immunoglobulin(Ig)M-CLIA, immunoglobulin(Ig)G-CLIA and IgM-IFA methods were 93.94%, 75.76%, and 83.64%, respectively. The positive likelihood ratio (PLR) and specificity of IgM-CLIA was 19.40 and 95.49%, respectively. The consistency coefficient of (IgM+IgG)-CLIA and PPA (titer ≥ 1:160) was 89.1%, and the sensitivity and negative predictive value of (IgM+IgG)-CLIA were 96.88% and 98.94%, respectively. CLIA MP-IgM has high concordance with PPA, and its specificity and sensitivity are higher than those of CLIA MP-IgG and IFA MP-IgM, suggesting its better diagnosis of early MP infection. The sensitivity and negative predictive value of CLIA MP (IgM+IgG) were higher than those of PPA or IFA, indicating that it should be considered as a priority in the diagnosis of MP infection.

## 1. Introduction

*Mycoplasma pneumoniae* is one of the most frequently detected pathogens of atypical pneumonia and is responsible for more than 20% of community-acquired pneumonia cases worldwide, especially in children and adolescents.^[1,2]^ It usually causes respiratory tract infections, even leading to death in severe cases.^[3–5]^ Early diagnosis is crucial for the immediate treatment of *M pneumoniae* infection. There are different diagnostic tests, but neither polymerase chain reaction (PCR) nor culture methods can distinguish colonization from symptomatic infections.^[6,7]^ Serologic assays such as passive particle agglutination (PPA) and immunofluorescence assay (IFA) for detecting serum *M pneumoniae*-immunoglobulin (Ig) antibodies are still the main laboratory methods for the clinical diagnosis of *M pneumoniae* infection. Currently, no single test can accurately identify *M pneumoniae* infection, and the combination of multiple detection methods is the most reliable approach,^[8,9]^ which can not only improve diagnostic specificity and sensitivity, but also reduce false-negative and false-positive rates. Serology tests are basic and important tools for the diagnosis of *M pneumoniae* infection.^[7]^ Chemiluminescence immunoassay (CLIA), a convenient operation and highly sensitive, has also been widely used for serological testing of *M pneumoniae* infections in recent years. In this retrospective study, 165 blood samples were collected to detect *M pneumoniae* infection using PPA, IFA, and CLIA, which were compared in their clinical application value for finding the most helpful method in the diagnosis and treatment of *M pneumoniae* infection.

## 2. Materials and methods

### 2.1. Study population

The study population consisted of 165 children (79 males and 86 females) with fever, cough, and/or radiologically confirmed 60 cases of bronchitis,^[10]^ 3 cases of bronchiolitis,^[11]^ 76 cases of bronchopneumonia, 10 cases of pneumonia^[12]^ and 16 cases of upper respiratory tract infection, including 15 outpatients and 150 inpatients who were admitted to Qilu Hospital (Qingdao) from March 1, 2019 to April 20, 2019. The range of patients was less than 14 years (range: 2 months–14 years, mean age 4.37 ± 3.01 years). Children with fever, cough, and/or radiologically confirmed respiratory tract infection were included in the study. The children with immunosuppressive illness, bronchial asthma, noninfectious pulmonary disease or who have had respiratory tract infections within 3 months were excluded. This study was approved by the Ethics Committee of Shandong University Qilu Hospital (Qingdao,KYLL-KS-2023130).

### 2.2. Methods

Blood sample in all children were collected for laboratory test including the PPA test, Chemiluminescent immunoassay and Immunofluorescence assay.

Blood samples were collected from 1 day after the onset of the disease at the earliest and 30 days after the onset of the disease at the latest, with an average of 5.10 ± 4.21 days. Serum was separated by centrifugation following clot retraction and tested immediately. Samples were stored at 4°C for less than 48 hours if they were not detected on time.

#### 2.2.1. Passive particle agglutination.

The PPA test (Serodia, Myco II, Fujirebio, Japan) was used to detect M. *M pneumoniae* antibodies, and the antigen labeled with manufactured artificial gelatin particles was the cell membrane component of *M pneumoniae* (Mac strain). Serum was diluted to a final reaction concentration of 1:40 to 1:20,480, and unsensitized particles were added to the control well with a final dilution of 1:20. The results were recorded after 3 hours. A definite compact button in the center of the well with a smooth round outer margin was read as a negative result, and a definite large ring with firmly agglutinated particles spread within the ring was read as a positive result. A titer of ≥1:40 was regarded as positive for *M pneumonia*e antibodies, and ≥1:160 was regarded as a recent or acute M. pneumoniae infection of *M pneumoniae*.

#### 2.2.2. Chemiluminescent immunoassay.

Serum IgM and IgG specific *M pneumoniae* antibodies were detected using 2-step direct chemiluminescence technique on YHLO iFlash 3000-A CLIA analyzer (YHLO Biotech, Shenzhen, China) according to the manufacturer’s instructions. IgG > 36 AU/mL and IgM > 1.1 Coi were judged as positive.

#### 2.2.3. Indirect immunofluorescence assay.

Serological testing for *M pneumoniae*-specific serum IgM was performed using the indirect immunofluorescence method (EUROIMMUN, Medizinische Labordiagnostika AG), according to the manufacturer’s instructions. Fluorescence was observed under a fluorescence microscope, *M pneumoniae* antigen on the biofilm combined with *M pneumoniae* IgM in the serum; net-like bright fluorescence was judged as positive. All samples were detected by experienced technicians.

### 2.3. Statistical analysis

Statistical analyses were performed using SPSS 20.0 version (SPSS, Chicago, IL). Quantitative data are presented as the mean ± SD and qualitative data are presented as percentages (cases). Chi-square tests were used to compare groups, *P* ≤ .05. The proportions were compared using the χ^2^ test. The kappa coefficient was calculated to compare the consistency of different methods. Kappa ≥ 0.75 indicates good consistency, 0.75 > kappa ≥ 0.4 for medium consistency, and kappa < 0.4 for poor consistency. Spearman’s tests were used to determine the correlation between age and positive rates of the 3 methods. *R*s > 0 indicated a positive correlation, *R*s **<** 0 indicated a negative correlation, and *R*s = 0 indicated no correlation. |*R*s| between 0.8–1.0 indicates a high strong correlation, 0.6–0.8 for strong correlation, 0.4–0.6 for moderate correlation, 0.2–0.4 for weak correlation, and 0.0–0.2 for no correlation. The higher Likelihood ratio indicate the accuracy of the diagnosis and LR^+^ >10 usually can be used for the diagnosis.^[13]^

## 3. Results

### 3.1. Detection Yield of different techniques

PPA was positive in 59 cases (titer ≥ 1:40), 32 of which had titers of ≥1:160. The IgM-IFA results were positive in 47 patients. 66 cases were IgG-CLIA-positive, 34 cases were IgM-CLIA-positive, 29 cases were (IgG+IgM)-CLIA positive and 71 patients were positive for *M pneumoniae* antibody IgG-CLIA and IgM-CLIA alone or simultaneously. Females had a higher positive rate of *M pneumoniae* antibodies than males using the 3 methods, but the difference was not significant (Table 1). Patients aged 6 to 13 years had the highest positivity rate for *M pneumoniae*. The Rs of the positive rate between IgG-CLIA and age was the highest at 0.443, while IgM-CLIA was the lowest at 0.17 (Table 2).

**Table 1:** Results of *M pneumoniae* positive of different genders.

Gender	N (%)	CLIA			IFA	PPA	
		IgG	IgM	IgG+IgM*	IgM	≥1:40	≥1:160
Male	79 (47.88)	29 (36.71)	14 (17.72)	32 (40.51)	19 (11.52)	24 (30.38)	12 (15.19)
Female	86 (52.12)	37 (43.02)	20 (23.26)	39 (45.35)	28 (16.97)	35 (40.70)	20 (23.26)
Total	165 (100.00)	66 (40.00)	34 (20.61)	71 (43.03)	47 (28.48)	59 (35.76)	32 (19.39)
	χ^2^	0.684	0.771	0.394	1.463	1.908	1.714
	*P*	.408	.380	.530	.226	.167	.191

CLIA = chemiluminescence immunoassay, IFA = indirect immunofluorescence assay, PPA = passive particle agglutination.

* IgG+IgM: positive alone or simultaneously.

**Table 2:** Results of *M pneumoniae* positive of different age.

Age (yr)	N (%)	CLIA			IFA	PPA	
		IgG	IgM	IgG+IgM*	IgM	≥1:40	≥1:160
<1	14 (8.48)	0 (0.00)	0 (0.00)	0 (0.00)	0 (0.00)	0 (0.00)	0 (0.00)
1–2	52 (31.52)	11 (21.15)	8 (15.38)	14 (26.92)	12 (23.08)	13 (25.0)	8 (15.38)
3–5	57 (34.55)	26 (45.61)	15 (26.32)	28 (49.12)	19 (33.33)	24 (42.11)	12 (21.05)
6–13	42 (25.45)	29 (69.05)	11 (26.19)	29 (69.05)	16 (38.10)	22 (52.38)	13 (30.95)
Total	165 (100.00)	66 (40.00)	34 (20.61)	71 (43.03)	47 (28.48)	59 (35.76)	33 (20.00)
	*R*s	0.443	0.17	0.411	0.207	0.303	0.201
	*P*	.000	.029	.000	.080	.000	.010

CLIA = chemiluminescence immunoassay, IFA = indirect immunofluorescence assay, PPA = passive particle agglutination.

* IgG+IgM: positive alone or simultaneously.

### 3.2. Comparison of CLIA and PPA

In the PPA titer ≥ 1:40 group (59 cases), 32, 53, and 56 patients were positive for IgM-CLIA, IgG-CLIA, and (IgM+IgG)-CLIA, respectively. The consistency coefficients are 82.4%, 88.5%, and 89.1%, respectively. Kappa coefficients were 0.578, 0.756, and 0.773, respectively. In the titer ≥1:160 group (32 cases), 28, 29 and 31 cases were positive for IgM-CLIA, IgG-CLIA, and (IgM+IgG)-CLIA, respectively. The consistency coefficients were 93.94%, 75.76%, and 75.2%, respectively. Kappa coefficients were 0.754, 0.448 and 0.457 respectively, the consistency coefficients between IgG-CLIA and PPA was significantly different with that between IgM-CLIA and PPA (χ^2^ = 21.21, *P* < .01) (Tables 3 and 4).

**Table 3:** The consistency of 3 methods.

Groups	Methods		Total patients (165)		
			N	Consistency coefficient %	Kappa
IFA and PPA	IgM-CLIA(−)	PPA(−)	99	84.2	0.641 (*P* = .029)
	IgM-CLIA(+)	PPA(+)	40		
	IgM-CLIA(−)	PPA(+)	19		
	IgM-CLIA(+)	PPA(−)	7		
CLIA and PPA	IgM-CLIA(−)	PPA(−)	104	82.4	0.578 (*P* = .000)
	IgM-CLIA(+)	PPA(+)	32		
	IgM-CLIA(−)	PPA(+)	27		
	IgM-CLIA(+)	PPA(−)	2		
	IgG-CLIA(−)	PPA(−)	93	88.5	0.756 (*P* = .167)
	IgG-CLIA(+)	PPA(+)	53		
	IgG-CLIA(−)	PPA(+)	6		
	IgG-CLIA(+)	PPA(−)	13		
	(IgM+IgG)-CLIA(−)	PPA(−)	91	89.1	0.773 (*P* = .008)
	(IgM+IgG)-CLIA(+)	PPA(+)	56		
	(IgM+IgG)-CLIA(−)	PPA(+)	3		
	(IgM+IgG)-CLIA(+)	PPA(−)	15		
	IgG-CLIA(−)	IgM-CLIA(−)	94	74.5	0.423 (*P* = .000)
	IgG-CLIA(+)	IgM-CLIA(+)	29		
	IgG-CLIA(+)	IgM-CLIA(−)	37		
	IgG-CLIA(−)	IgM-CLIA(+)	5		
IFA and CLIA	IgM-IFA(−)	IgM-CLIA(−)	112	84.8	0.594 (*P* = .015)
	IgM-IFA(+)	IgM-CLIA(+)	28		
	IgM-IFA(−)	IgM-CLIA(+)	6		
	IgM-IFA(+)	IgM-CLIA(−)	19		

CLIA = chemiluminescence immunoassay, IFA = indirect immunofluorescence assay, PPA = passive particle agglutination.

*IgG+IgM: positive alone or simultaneously.

**Table 4:** The comparation of CLIA and IFA with PPA (≥1:160).

Methods	PPA (≥1:160)			Total	Sensitivity	Specificity	PLR	PPV	NPV	Concordance rate	Kappa	χ^2^
	+		−									
IgM-CLIA	+	28	6	34	87.50%	95.49%	19.4	82.35%	96.95%	93.94%*	0.754 (*P* = .811)	21.21
	−	4	127	131								
Total		32	133	165								
IgG-CLIA	+	29	37	66	90.63%	72.18%	3.25	43.94%	96.97%	75.76%	0.448 (*P* = .000)	
	−	3	96	99								
Total		32	133	165								
(IgM+IgG)-CLIA	+	31	40	71	96.88%	69.92%	3.22	43.66%	98.94%	75.2%	0.457 (*P* = .000)	
	−	1	93	94								
Total		32	133	165								
IgM-IFA	+	26	21	47	81.25%	84.21%	5.15	55.32%	94.92%	83.64%*	0.556 (*P* = .006)	8.797*
	−	6	112	118								
Total		32	133	165								

CLIA = chemiluminescence immunoassay, IFA = indirect immunofluorescence assay, NPV = negative predictive value, PLR = positive likelihood ratio, PPA = passive particle agglutination, PPV = positive predictive value.

* IgM-CLIA compared with IgM-IFA. IgG+IgM: positive alone or simultaneously.

### 3.3. IFA compared with PPA and CLIA

Forty cases were positive for IgM-CLIA and PPA (titer ≥ 1:40) (consistency coefficient 84.2%, kappa = 0.641), 26 of which were positive for PPA ≥ 1:160 (consistency coefficient 83.64%, kappa = 0.556). The consistency coefficients of IgM-CLIA and PPA ≥ 1:160 were significantly different from those of IgM-IFA and PPA ≥ 1:160 (χ^2^ = 8.797, *P* < .01). Twenty-eight cases were positive in the IgM-CLIA and IgM-IFA tests (consistency coefficients 84.8%, kappa = 0.594) (Tables 3 and 4).

## 4. Discussion

*Mycoplasma pneumoniae* pneumonia is one of the most common infections in children with community-acquired pneumonia around the world. The incidence and prevalence of *M pneumoniae* infection are different with periods, regions and populations. There were variations in the positive rate of *M pneumoniae* infection according to age; it was usually high for children and adolescents^[1,2,8,14,15]^ and low for infants and adults.^[2,8,16–18]^ For the child population, most studies have reported a higher positive rate for children aged > 5 years than for those < 5 years old^[1,2,18–20]^; the same result was observed in our study, which showed that children under the age of 6 years had the highest infection rate, which is thought to be related to incomplete immune mechanisms in this group and was easily infected by *M pneumoniae*.^[5,21]^ Some studies have shown sex differences in *M pneumoniae* infections,^[1,5,22,23]^ with some showing no differences.^[16,18]^ In this study, no difference was observed; although the positive rates of females were higher than those of males in all 3 methods, the difference was not statistically significant. We believe that these differences require further research and data for analysis and confirmation.

Accurate detection is essential for the diagnosis and treatment of *M pneumoniae* infections; however, to date, there is no ideal method. *M pneumoniae* culture is usually treated as the golden diagnostic criterion for *M pneumoniae* infection,^[24]^ but it is insensitive and time-consuming, and is not recommended for routine assays.^[7,25]^ PCR was used early in 1989 to detect *M pneumoniae*, as it was more sensitive and rapid,^[26]^ but no matter culture or PCR couldn’t distinguish healthy subjects from suspected cases of *M pneumoniae* infection.^[6,25]^ According to previous studies, *M pneumoniae* can be isolated from throat swabs and cause asymptomatic infections in healthy children.^[6]^ In addition, the results of culture and PCR are greatly influenced by factors such as sample collection and contamination, so neither of them can replace serologic assays.^[25,27]^ Serological tests, such as PPA and IFA, are still the most widely used detection methods for *M. pneumoniae* in clinical practice. Serological assays for specific antibodies against *M pneumoniae* include the detection of IgM and IgG antibodies. *M pneumoniae* IgM could be detected in the first week and reached peak titers in the third week after *M pneumoniae* infection, *M pneumoniae* IgG usually appears 2 weeks after the onset of *M pneumoniae* infection. In clinical *M pneumoniae* antibody titers ≥ 1:160 detected by PPA were regarded as a reference for the diagnosis of *M pneumoniae* recent or acute M. pneumoniae infection.^[24]^ Acute and convalescent phase sera immunoglobulinantibody titers of *M pneumoniae* had a 4-fold rise or decrease in the diagnosis *M pneumoniae* infection.^[7,24]^ As *M pneumoniae* IgG may last several years in the blood of patients after infection, and PPA detection cannot differentiate the antibody classes, it is time consuming and may delay prophylaxis and treatment. In addition that the results of PPA were easily influenced by the operator’s subjective and environment factors, with the addition of the difficulty in obtaining convalescent blood sample from children in a short time, for the reasons above, the clinical value of PPA test especially sera Ig-antibody of *M pneumoniae* titers had 4-fold rise or decrease is limited. *M pneumoniae* IgM can be detected in sera early in the week after infection, and its half-life is short. Several studies have indicated that a single positive test can be used as a reference for recent *M pneumoniae* infections, and the detection of *M pneumoniae* IgM has been used for the early diagnosis and treatment of *M pneumoniae* infections.^[8,14,18,24]^

The CLIA method uses an automatic instrument; it is fast and flexible, and detection can be completed in 30 min. The sensitivity and specificity were better than those of IFA and PPA, the repeatability of the detection results was good, and IgM-CLIA and IgG could be detected separately.^[28]^ In our study, using PPA ≥ 1:160 as the standard, both the consistency coefficient and specificity of IFA-IgM were significantly lower than those of CLIA-IgM. The positive prediction rate of IFA-IgM was only 55.32%, whereas that of CLIA-IgM was 96.88%. Our data showed that the concordance rate between IgM-CLIA and PPA ≥ 1:160 was higher than that between IgG-CLIA and IFA, with a sensitivity of 95.49%, indicating good clinical application value for the diagnosis and treatment of *M pneumoniae* infections. We thought that 1 reason for the concordance rate between IgG-CLIA and PPA ≥ 1:160 lower than IgM-CLIA was that IgG antibody is a monomer with only 2 antigen-binding sites, so it needs a high concentration to bind to the particle antigen to show positive results. IgM antibody is a pentamer with a J-chain and theoretically has ten antigenic binding sites; it can combine with the corresponding antigen and form a huge grid-like structure at a relatively low concentration and showed a higher positive rate. Beersma et al^[29]^ thought that the combined detection of IgG and IgM can enhance substantially higher diagnostic yields than assays for IgM alone, especially in patients with weak IgM responses after *M pneumoniae* infection. In addition, IgM-CLIA combined with IgG-CLIA detection can improve the accuracy of detection of *M pneumoniae* infections and prevent overdiagnosis. In our study, the consistency coefficients between *M pneumoniae* (IgG+IgM)-CLIA and PPA were 89.1% higher than 79.6% reported by Chen et al.^[30]^ This may be related to the differences in the enrolled patients and needs to be further verified by clinical application.

There were some limitations to our study, such as the small sample size and the fact that all samples were collected in less than 2 months from March to April in 2019, which may have resulted in the loss of the prevalence of *pneumoniae* infection. Additionally, we did not perform a PCR assay for comparison with the CLIA method in this study, which showed that more samples and populations need to be included in our next step.

## 5. Conclusions

*Mycoplasma pneumoniae* IgM-CLIA has a high concordance with PPA, and its specificity and sensitivity are higher than those of IgG-CLIA and IFA, suggesting that it can accurately indicate early infection and help doctors treat *M pneumoniae* infection in the early time. The sensitivity and negative predictive value of *M pneumoniae* (IgM+IgG)-CLIA were higher than those of PPA and IFA, which is useful for reducing the rate of misdiagnosis, especially in patients with a low concentration of *M pneumoniae* IgM. Compared with manual detection methods such as PPA and IFA, automated chemiluminescence with the advantages of higher sensitivity, specificity, and accuracy should be widely applied for the laboratory diagnosis of *M pneumoniae* infection.

## Author contributions

**Conceptualization:** Mengyang Liu, Ke Meng, Shiying Sun.

**Data curation:** Mengyang Liu, Ke Meng, Jun Jiang.

**Methodology:** Mengyang Liu, Ke Meng, Jun Jiang.

**Project administration:** Mengyang Liu, Ke Meng, Jun Jiang, Shiying Sun.

**Software:** Mengyang Liu.

**Supervision:** Ke Meng, Jun Jiang.

**Writing – original draft:** Mengyang Liu.

**Writing – review & editing:** Mengyang Liu, Li Zhang, Shiying Sun.
